# Sphingolipid metabolism in colorectal adenomas varies depending on histological architecture of polyps and grade of nuclear dysplasia

**DOI:** 10.1007/s11745-014-3987-3

**Published:** 2015-01-17

**Authors:** Krzysztof Kurek, Bartłomiej Łukaszuk, Agnieszka Świdnicka-Siergiejko, Paweł Rogalski, Eugeniusz Wróblewski, Adrian Chabowski, Andrzej Dąbrowski, Małgorzata Żendzian-Piotrowska

**Affiliations:** 1 Department of Physiology, Medical University of Bialystok, ul. Mickiewicza 2C, 15-222 Białystok, Poland; 2 Department of Gastroenterology and Internal Medicine, Medical University of Bialystok, ul. M. Sklodowskiej-Curie 24A, 15-276 Białystok, Poland

**Keywords:** Ceramide, Colorectal adenomas, Colorectal cancer, Sphingolipids, Sphingosine-1-phosphate

## Abstract

Incidence of colorectal cancer (CRC) is growing worldwide. Pathogenetic mechanisms responsible for its onset and progression need further clarification. Colorectal adenomatous polyps are precancerous lesions with malignant potential dependent on histological architecture and grade of nuclear dysplasia. One of the factors conditioning CRC development are abnormalities in sphingolipid metabolism. The aim of this study was to assess the levels of sphingolipids in human colorectal adenomas. The control group (C, *n* = 12) consisted of patients with no colonic polyps. The examined group consisted of patients with prior diagnosed colonic polyps, qualified to endoscopic polypectomy. This group was further divided due to histological architecture into tubular adenomas group (TA, *n* = 10), tubulovillous adenomas with low-grade dysplasia (LGD-TVA, *n* = 10), and tubulovillous adenomas group with high-grade dysplasia (HGD-TVA, *n* = 11). In tissue samples, sphingolipd metabolite contents were measured using high performance liquid chromatography (HPLC). In cases of polypoid lesions with low malignancy potential (tubular adenomas), concentration of ceramide, which is characterized by proapoptotic and anti-proliferative properties, increases compared with control group (*p* < 0.05), whereas content of sphingosine-1-phosphate with anti-apoptotic and stimulating cellular proliferation properties is reduced in comparison with control group (*p* < 0.05). On the contrary, in cases of more advanced form of adenomatous polyps (tubulovillous adenomas with high-grade dysplasia), the ceramide level decreases compared with control group (*p* < 0.05) while sphingosine-1-phosphate concentration is elevated (*p* < 0.05). We found that concentrations of pro-apoptotic ceramide are decreased and pro-proliferative S1P levels are increased in polypoid lesions with high malignancy potential, and it was the opposite in those with low malignancy potential.

## Introduction

Colorectal cancer (CRC) is the third most common cancer in Western countries, and incidence of this neoplasm is growing. The World Health Organization (WHO) recently reported morbidity of CRC—9.4 % of women and 10 % of men worldwide [1]. Although it is estimated that 75–95 % of CRCs develop in patients with moderate or no genetic risk [2], there are a number of genetic syndromes, including familial adenomatous syndrome (FAP), Lynch Syndrome, and Gardner Syndrome which are associated with higher rates of CRC [3, 4]. Moreover, familial history increases probability of incidence of this disease and it is estimated that patients with a familial history of CRC in two or more first-degree relatives have even a threefold greater risk of this cancer [5]. Patients with inflammatory bowel disease (IBD), such as ulcerative colitis and Crohn’s disease are also at increased risk of CRC [6]. Other risk factors include age, male sex, dietary habits (alcohol consumption, diet rich in fat, red or processed meat, low intake of fruits and vegetables), obesity, long-term smoking, and a lack of physical activity [7].

Colorectal adenomas (CRAs) are premalignant lesions, occurring as a result of a gut mucosal proliferation. It was estimated that more than 80 % of colorectal cancers arise from colorectal adenomas. Based on histological architecture, colorectal adenomas can be divided into tubular adenomas (TAs), villous adenomas (VAs), and tubulovillous adenomas (TVAs) which have a mixture of tubular and villous compounds [8]. VAs are considered to bring a higher risk of malignant transformation (40 % risk of cancer) than TAs (5 % risk of cancer) or TVAs (20 % risk of cancer) [9]. Besides histological architecture, another important factor of cancerous potential is lesion size. Although adenomas with a diameter <1 cm have very low potential for malignancy, lesions >2 cm are characterized by 20 % risk of malignant transformation. However, VAs have similar malignant potential as TAs and TVAs if sizes of the lesions are comparable [10]. Regardless of lesion size and histological architecture each polyp can undergo malignant transformation. Thus, interruption of adenoma-carcinoma sequence via polypectomy (endoscopic removal) reduces CRC incidence [11].

Sphingolipids constitute a group of lipid organic molecules, composed of a sphingoid base and free fatty acids residues. The main route of sphingolipid synthesis is by a de novo pathway, where the first reaction, catalyzed by serine palmitoyltransferase (SPT), is condensation of amino acid serine with palmitoyl-CoA. The product of this reaction, 3-ketosphinganine, is further reduced to sphinganine (SFA) and finally acylated to dihydroceramide. In the last step of the de novo pathway dihydroceramidase desaturase (ceramide synthase) introduces a double bond between C4 and C5 position in sphingoid base to form ceramide. Another important route of ceramide synthesis is hydrolysis of membrane sphingomyelin (SM) catalyzed by sphingomyelinase (SMase). Ceramide degradation by ceramidases results in formation of sphingosine (SFO). Sphingosine-1-phosphate (S1P) is another bioactive molecule generated by SFO phosphorylation, reaction catalyzed by sphingosine kinase (SPHK) [12]. Bioactive sphingolipids (ceramide, SFA, SFO, S1P) regulate and modulate numerous physiological and pathophysiological processes including cellular growth and differentiation, apoptosis, angiogenesis, and inflammatory response [13]. It is very important to emphasize at this point that ceramide and its derivatives usually exert opposing effects in the cell. Synthesized ceramide (de novo or through SM hydrolysis) has been shown to have anti-proliferative and proapoptotic properties [14]. It can be postulated that ceramide, via inhibition of cellular proliferation and differentiation, diminishes growth of neoplasmatic cells and consequently inhibits tumorigenesis [15]. On the other hand, S1P promotes cell proliferation and inhibits apoptosis [13]. These properties may enhance cellular proliferation, differentiation, and increase angiogenesis. In contrast to ceramide, increased S1P level was revealed in many types of neoplasms [16].

The importance of sphingolipids in CRC progression is considered since 1986 when Dudeja et al. [17] demonstrated that rats treated with chemical carcinogen 1,2-dimethylhydrazine were characterized by significantly increased SM level in colonic tissue [17]. Another study showed that dietary SM supplementation in mice prevented formation of aberrant crypt foci in colonic mucosa [18]. These early findings derived from animal models revealed that ceramide precursor SM counteracts CRC development. On the other hand, study by Selzner et al. [19] demonstrated that in human colon cancer tissue, levels of SM and ceramide were both decreased compared with normal colon mucosa [19]. Presented changes in sphingolipid levels are secondary to alterations in activities of enzymes regulating their metabolism. S1P has also an impact on colorectal carcinogenesis. Increased levels of this molecule have been demonstrated in both human CRC tissue and in animals treated with carcinogenic azoxymethane [20]. Moreover, by using antibodies against S1P, inhibition of growth, invasion, and angiogenesis in CRC could be obtained [21]. The main enzyme responsible for S1P formation is sphingosine kinase. In the APC^Min/+^ mouse (animal model of FAP) Kohno et al. [22] proved that knock-out of this enzyme results in decreased intracellular S1P level followed by a reduction in adenomas size and significant inhibition of cellular proliferation [22].

To summarize above data it can be concluded that sphingolipids play an important role in CRC onset and development. However, metabolism of sphingolipids in precancerous colorectal adenomas has so far been poorly investigated. Therefore, the aim of this study was to assess the levels of sphingolipids, as well as the activities of enzymes which regulate their metabolism, in human CRAs.

## Materials and methods

### Subjects

The investigation was approved by the Ethics Committee for Human Studies of the Medical University of Bialystok (no. R-I-002/499/2012). The study enrolled patients hospitalized at the Department of Gastroenterology and Internal Medicine from September 2012 to November 2013. All patients gave their written consent for participation in this project. The control group (C, *n* = 12) consisted of patients in whom no abnormalities in large bowel during colonoscopy were observed. The other group consisted of patients with prior diagnosed colonic polypoid lesions, qualified to endoscopic polypectomy. This group was further divided due to histological architecture of the polyp into a tubular adenomas group (TA, *n* = 10), tubulovillous adenomas with low-grade dysplasia (LGD-TVA, *n* = 10), and a tubulovillous adenomas group with high-grade dysplasia (HGD-TVA, *n* = 11). All groups were homogeneous with regard to patients' gender, age, and body mass index (BMI). Subjects with concomitant diseases affecting sphingolipid metabolism (diabetes, obesity, metabolic syndrome) were excluded from further evaluation. Patients with prior history of any neoplastic diseases and abdominal surgery were also excluded.

### Serum analyses

Fasting blood glucose level was measured with an Accu-check (Bayer, Germany) glucose meter. Blood samples were obtained from the fingertip.

Plasma cholesterol, triacylglycerol (TG), high-density lipoprotein (HDL), and carcino-embryonic antigen (CEA) content were measured with commercially available kits (Abbot, USA). Blood samples were obtained from the ulnar vein.

### Endoscopic procedure

All colonoscopies were performed by the same experienced endoscopist after typical preparation (4 L of polyethylene glycol taken in two split doses). Mucosa samples were obtained using biopsy forceps. In each subject from the control group approximately 16 mg of tissue was collected from normal mucosa (4 mg per each of four biopsy replicates). In each subject from the studied groups about 16 mg of tissue was obtained from polypoid lesions (4 mg per each of four biopsy replicates) immediately before endoscopic polypectomy. Tissue samples were freeze-clamped with aluminum tongs precooled in liquid nitrogen and stored at −80 °C until further analyses. Removed polypoid lesions were transferred for histological evaluation. No early or delayed complications related to tissue sampling or endoscopic polypectomy were noticed.

### Ceramide content

Ceramide content was measured as previously described [23]. A 50-µl volume of the chloroform phase containing lipids extract was transferred to a fresh tube containing an internal standard [C17-sphingosine, (Avanti Polar Lipids, UK)]. Samples were next evaporated under a nitrogen stream, redissolved in 1.2 ml of 1 M KOH in 90 % methanol and heated at 90 °C for 60 min to convert ceramide into sphingosine. Samples were then partitioned by the addition of chloroform and water and the upper phase was discarded. The lower phase was evaporated under nitrogen and then redissolved in ethanol. The content of free sphingosine, liberated from ceramide, was next analyzed by means of a high performance liquid chromatography (HPLC) system (ProStar, Varian Inc.) equipped with a fluorescence detector and C18 reverse-phase column (Varian Inc., OmniSpher 5, 4.6 × 150 mm). Using *N*-palmitoylsphingosine (Avanti Polar Lipids, UK) as a standard, a calibration curve was prepared. The chloroform extract used for the analysis of ceramide level also contains small amounts of free sphingoid bases. Therefore, the content of ceramide was corrected for the level of free sphingosine determined in the same sample.

### Sphingomyelin content

Sphingomyelin content was measured as previously described [23]. Frozen tissue samples were pulverized in precooled aluminum mortar. The powder was then transferred to a tube containing methanol and 0.01 % butylated hydroxytoluene (Sigma) as an antioxidant. Lipid fractions were next extracted using the method described by Bligh and Dyer [24]. Sphingomyelin was isolated by means of thin-layer chromatography (TLC). Briefly, gel bands, corresponding to the standard, were scraped off the plates and transferred into screw-cap tubes which contained pentadecanoic acid (Sigma–Aldrich, USA) as an internal standard. Sphingomyelin fatty acids were then transmethylated and subsequently analyzed by means of gas-liquid chromatography (GLC). A Hewlett-Packard 5890 Series II system, equipped with a double flame ionization detector and Agilent CP-Sil 88 capillary column (100 m, internal diameter of 0.25 mm), were used. The content of sphingomyelin is presented as the sum of individual fatty acid species of the assessed fraction.

### Sphinganine, sphingosine and sphingosine-1-phosphate contents

Sphinganine, sphingosine, and sphingosine-1-phosphate contents were measured according to the method, described by Min et al. [25]. Internal standards [C17-sphingosine and C17-S1P, (Avanti Polar Lipids, USA)] were added prior to sample homogenization and ultra-sonication. The sphingoid bases were converted to their *o*-phthalaldehyde derivatives and analyzed on an HPLC system (ProStar, Varian, Inc., USA) equipped with a fluorescence detector and C18 reverse-phase column (Varian, Inc., OmniSpher 5, 4.6 mm × 150 mm).

### Western blotting

Serine palmitoyltransferase, alkaline sphingomyelinase, and sphingosine kinase 1 expressions were determined using Western blot method. Routine Western blotting procedures were used to detect protein content as described previously [23]. The cells were lysed in ice-cold RIPA (radioimmunoprecipitation assay) buffer (50 mM Tris–HCl, 150 M NaCl, 1 mM EDTA, 1 % NP-40, 0.25 % Na-deoxycholate, 1 mM phenylmethylsulfonyl fluoride, 1 µg/ml aprotinin, 1 µg/ml leupeptin, 1 µg/ml pepstatin, 1 mM sodium orthovanadate, 1 mM sodium fluoride) and sonicated for 1 min at 4 °C. Protein concentration was determined using a BCA protein assay kit with bovine serum albumin as a standard. Samples were boiled at 95 °C for 10 min in sample buffer containing 2-mercaptoethanol. Protein (60 µg) was subjected to SDS-PAGE and transferred to PVDF membranes, followed by blocking membranes in TTBS buffer (50 mM Tris–HCl, 130 mM NaCl and 0.05 % Tween-20) containing 5 % nonfat dry milk for 90 min at room temperature. The membranes were then incubated overnight at 4 °C with the corresponding antibodies at a dilution of 1:500. Used primary antibodies were anti-SPHK1 (sc-48825, Santa Cruz Biotechnology, USA), anti-SMase (sc-49352, Santa Cruz Biotechnology, USA), and anti-SPT (SAB1300663, Sigma-Aldrich, USA). Thereafter, the membranes were incubated with anti-rabbit IgG horseradish peroxidase-conjugated secondary antibody at a dilution of 1:3,000 (sc-2004; Santa Cruz Biotechnology, USA). Immunoreactive protein bands were visualized using an enhanced chemiluminescence substrate (Thermo Scientific, USA) and quantified by densitometry (Biorad, USA). Equal protein concentrations were loaded in each lane as confirmed by Ponceau staining of the blot membrane. Protein expression was normalized for β-tubulin and reported as arbitrary units. Finally, the control was set to 100 % and the experimental groups were expressed relative to the control [23].

### Statistical analysis

All data were visually inspected prior to analysis. The existence of possible outliers was tested via Grubbs' test. Identified outliers were verified for possible technical/transcription errors (e.g. tissue collection and processing). As a result of these operations, in total, three data points were excluded (≤10 % of data in the group; ≤7 % of all data points) from further analysis.

Results are shown as mean ± standard deviation (SD). Statistical differences between groups were assessed using ANOVA with a subsequent post hoc test (Tukey HSD) after log transformation of data (where appropriate). Statistical significance was set at *p* < 0.05.

## Results

### Subjects (Table 1)

Forty-one patients with previously diagnosed polypoid lesions in the colon were initially enrolled to investigation. Among them, nine were excluded because of concomitant diseases which could affect sphingolipid metabolism, and one refused to participate in the study. Finally, 31 patients with polypoid lesions in the colon and 12 patients from the control group were enrolled for further evaluation. Histological assessment identified 10 tubular adenomas, 10 tubulovillous adenomas with low-grade dysplasia, and 11 tubulovillous adenomas with high-grade dysplasia. Characteristics of each group are presented in Table 1. There were no significant differences in patients' gender, age, body mass index, or blood glucose level among all examined groups. Moreover, there were no significant differences in plasma cholesterol, TG, or HDL concentrations between examined groups. Compared with the C group, there was no significant difference in carcino-embryonic antigen concentration in the TA or LGD-TVA groups. However, the HGD-TVA group was characterized by significantly increased CEA content in comparison with the C (*p* < 0.01) group as well as with th TA (*p* < 0.01) and LGD-TVA (*p* < 0.01) groups.

**Table 1:** Characteristics of the subjects

Variable	C (*n* = 12)	TA (*n* = 10)	LGD-TVA (*n* = 10)	HGD-TVA (*n* = 11)
Sex	4 M, 4F	3 M, 4F	3 M, 4F	5 M, 3F
Age (years)	58.5 ± 7.4	50.3 ± 8.9	55.2 ± 6.6	58.2 ± 8.1
BMI (kg/m^2^)	24.4 ± 4.3	22.9 ± 3.2	25.7 ± 1.9	26.3 ± 5.0
Symptoms of IBD	No	No	No	No
Blood glucose (mg%)	89.3 ± 21.4	92.4 ± 15.3	95.0 ± 10.6	90.7 ± 18.5
Serum cholesterol (mg/dl)	165.3 ± 23.8	178.4 ± 15.6	159.2 ± 28.5	182.6 ± 18.4
Serum TG (mg/dl)	148.3 ± 26.3	162.1 ± 19.3	155.5 ± 23.9	160.3 ± 17.3
Serum HDL (mg/dl)	47.3 ± 13.9	50.4 ± 18.7	42.4 ± 17.3	52.1 ± 15.8
Serum CEA (μg/l)	4.5 ± 0.7	5.3 ± 1.2	4.9 ± 0.9	16.4 ± 2.6*^, #,†^

*BMI* body mass index, *TG* triacylglycerol, HDL high-density lipoprotein, CEA- carcino-embryonic antigen, *F* female gender, *M* male gender, *C* control group, *TA* tubular adenomas group, *LGD*-*TVA* tubulovillous adenomas with low-grade dysplasia group, *HGD*-*TVA* tubulovillous adenomas with high-grade dysplasia group, *IBD* inflammatory bowel diseases

The results are mean ± SD; * p < 0.05 compared with C; ^#^ *p* < 0.05 compared with TA; ^†^ *p* < 0.05 compared with LG-TVA

### Sphinganine content (Fig. 1)

Compared with C, the TA group was characterized by significant reduction of SFA concentration (*p* < 0.05). On the contrary, in LGD-TVA and HGD-TVA, a significant increase in SFA level was observed in comparison with the C group (*p* < 0.05 and *p* < 0.05, respectively). Further analysis revealed that in the LGD-TVA group, SFA level was significantly higher than in the TA group (*p* < 0.05). Finally, the HGD-TVA group was characterized by significant increase in SFA content compared with both TA and LGD-TVA groups (*p* < 0.05 and *p* < 0.05, respectively).

**Fig. 1:**
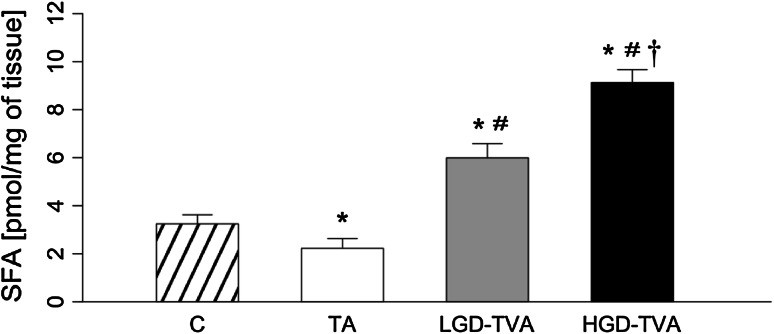
Comparison of sphinganine (SFA) content within all examined groups. *C* control group, *TA* tubular adenomas group, *LGD-TVA* tubulovillous adenomas with low-grade dysplasia group, *HGD-TVA* tubulovillous adenomas with high-grade dysplasia group. The results are mean ± SD. Results were back-transformed. *p < 0.05 compared with C group;^ #^ *p* < 0.05 compared with TA group; ^†^ *p* < 0.05 compared with LGD-TVA group

### Ceramide content (Fig. 2)

In comparison with the C group, both TA and LGD-TVA groups were characterized by a significant increase of ceramide concentration (*p* < 0.05 and *p* < 0.05, respectively). On the other hand, in the HGD-TVA group, a significant reduction of ceramide content, compared with the C group, was found (*p* < 0.05). The subgroup analysis demonstrated that in the LGD-TVA group, in comparison with the TA group, a significant decrement in ceramide content was noticed (*p* < 0.05). Whereas in HGD-TVA, a significant reduction of ceramide level was observed compared with both the TA and LGD-TVA groups (*p* < 0.05 and *p* < 0.05, respectively).

**Fig. 2:**
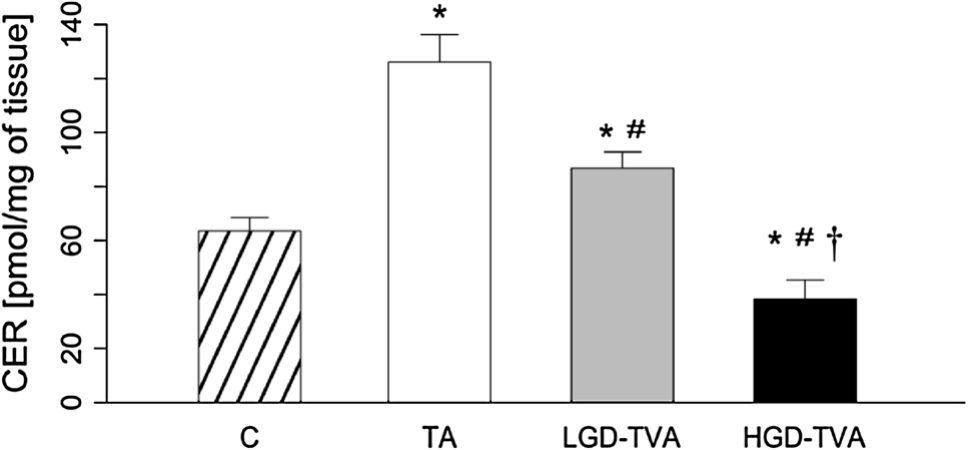
Comparison of ceramide (CER) content within all examined groups. *C* control group, *TA* tubular adenomas group, *LGD-TVA* tubulovillous adenomas with low-grade dysplasia group, *HGD-TVA* tubulovillous adenomas with high-grade dysplasia group. The results are mean ± SD; *p < 0.05 compared with C group;^ #^ *p* < 0.05 compared with TA group; ^†^ *p* < 0.05 compared with LGD-TVA group

### Sphingomyelin content (Fig. 3)

In relation to the C group, in the TA group, a significant decrement in SM concentration was observed (*p* < 0.05). In contrast with previous findings, the LGD-TVA and HGD-TVA groups were both characterized by a significant increase in SM level in comparison with the C group (*p* < 0.05 and *p* < 0.05, respectively). Compared with the TA group, the LGD-TVA group was characterized by a significant increase in SM content (*p* < 0.05). Finally, comparison of the HGD-TVA group with the TA group and LGD-TVA group has shown significant growth in SM concentration (*p* < 0.05 and *p* < 0.05, respectively).

**Fig. 3:**
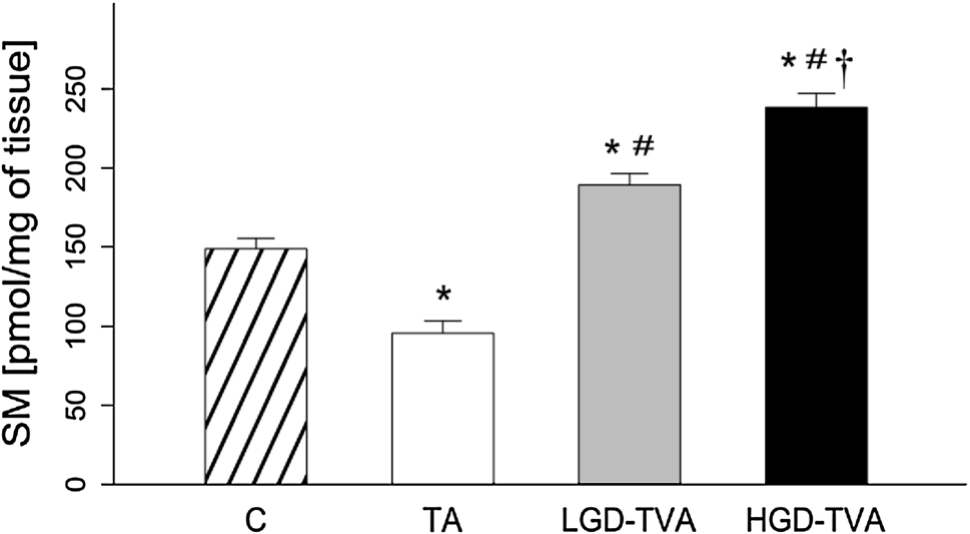
Comparison of sphingomyelin (SM) content within all examined groups. *C* control group, *TA* tubular adenomas group, *LGD-TVA* tubulovillous adenomas with low-grade dysplasia group, *HGD-TVA* tubulovillous adenomas with high-grade dysplasia group. The results are mean ± SD; *p < 0.05 compared with C group;^ #^ *p* < 0.05 compared with TA group; ^†^ *p* < 0.05 compared with LGD-TVA group

### Sphingosine content (Fig. 4)

Compared with C, all three groups, TA, LGD-TVA, and HGD-TVA, were characterized by a significant increment in SFO concentration (*p* < 0.05, *p* < 0.05, and *p* < 0.05, respectively). Further subgroup analysis demonstrated that only the HGD-TVA group was characterized by significant growth in SFO level in comparison with both the TA and LGD-TVA groups (*p* < 0.05 and *p* < 0.05, respectively). Interestingly, no significant differences between the LGD-TVA and TA groups were noticed.

**Fig. 4:**
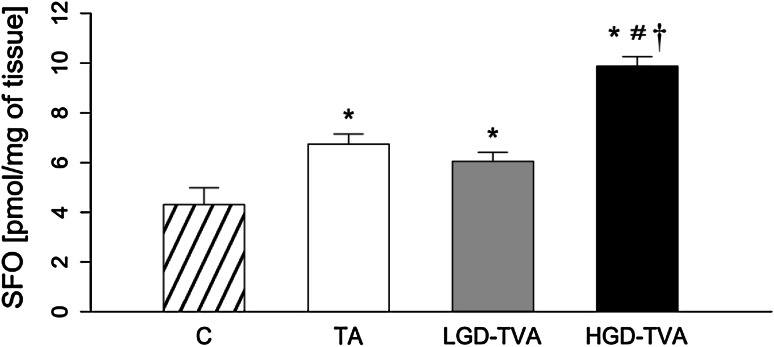
Comparison of sphingosine (SFO) content within all examined groups. *C* control group, *TA* tubular adenomas group, *LGD-TVA* tubulovillous adenomas with low-grade dysplasia group, *HGD-TVA* tubulovillous adenomas with high-grade dysplasia group. The results are mean ± SD. Results were back-transformed. *p < 0.05 compared with C group;^ #^ *p* < 0.05 compared with TA group; ^†^ *p* < 0.05 compared with LGD-TVA group

### Sphingosine-1-phosphate content (Fig. 5)

Compared with the C group, the TA and LGD-TVA groups were both characterized by significant reduction in S1P concentration (*p* < 0.05 and *p* < 0.05, respectively). On the contrary, a significant increase in S1P level was found (*p* < 0.05) between the HGD-TVA and C groups. Further analysis revealed that the LGD-TVA group was characterized by significant growth in S1P content in comparison with the TA group (*p* < 0.05). Finally, comparing the HGD-TVA group with the TA group and LGD-TVA group revealed a significant increment in S1P concentration (*p* < 0.05 and *p* < 0.05, respectively).

**Fig. 5:**
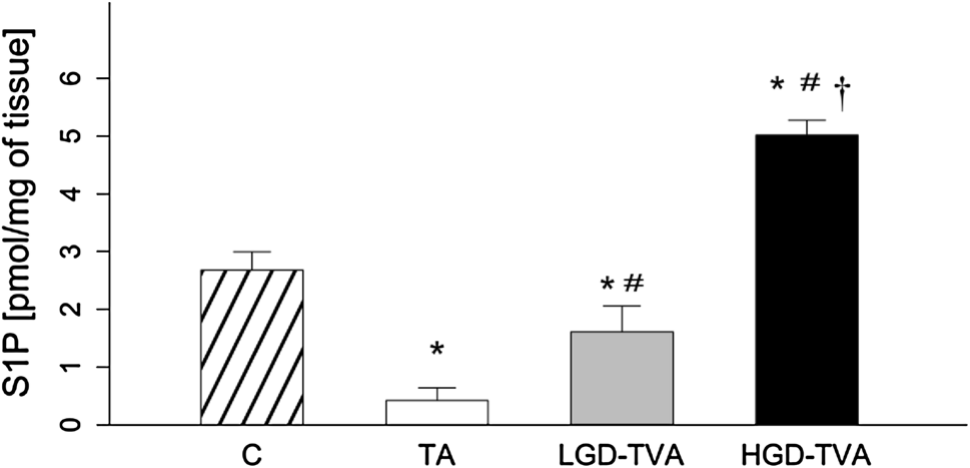
Comparison of sphingosine-1-phosphate (S1P) content within all examined groups. *C* control group, *TA* tubular adenomas group, *LGD-TVA* tubulovillous adenomas with low-grade dysplasia group, *HGD-TVA* tubulovillous adenomas with high-grade dysplasia group. The results are mean ± SD. Results were back-transformed. *p < 0.05 compared with C group;^ #^ *p* < 0.05 compared with TA group; ^†^ *p* < 0.05 compared with LGD-TVA group

### Serine palmitoyltransferase enzymatic protein expression (Fig. 6)

Expression of SPT enzymatic protein in the TA group was significantly lower in comparison with the C group (*p* < 0.05) which suggests decreased SPT activity in the TA group. On the contrary, expression of described enzymatic protein was significantly higher in both LGD-TVA and HGD-TVA compared with the C group (*p* < 0.05 and *p* < 0.05, respectively) suggesting increased SPT activity in both the LGD-TVA and HGD-TVA groups. The subgroup analysis showed that expression of SPT enzymatic protein was significantly higher in the LGD-TA group compared with the TA group (*p* < 0.05) suggesting increased SPT activity in the LGD-TA group. The HGD-TVA group was characterized by significant up-regulation of SPT enzymatic protein expression in comparison with both the TA and LGD-TVA groups (*p* < 0.05 and *p* < 0.05, respectively), which suggests increased SPT activity in both the TA and LGD-TVA groups.

**Fig. 6:**
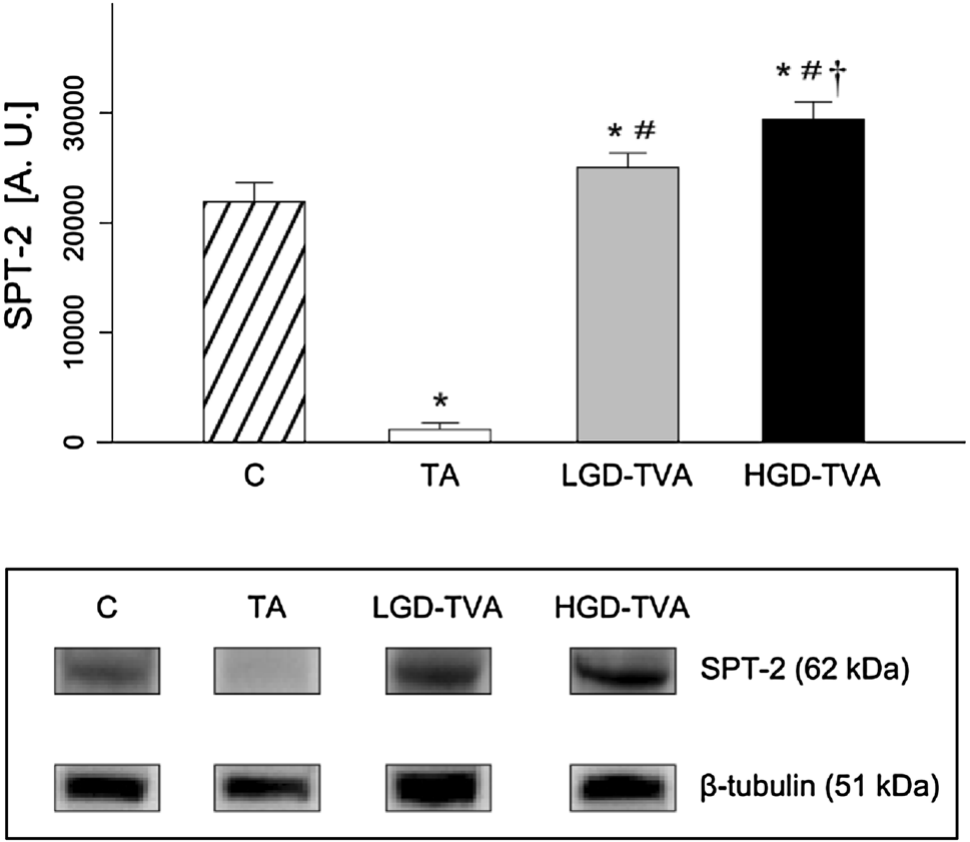
Comparison of serine palmitoyltransferase (SPT2) enzymatic protein expression within all examined groups. *C* control group, *TA* tubular adenomas group, *LGD-TVA* tubulovillous adenomas with low-grade dysplasia group, *HGD-TVA* tubulovillous adenomas with high-grade dysplasia group. The results are mean ± SD. Results were back-transformed. *p < 0.05 compared with C group;^ #^ *p* < 0.05 compared with TA group; ^†^ *p* < 0.05 compared with LGD-TVA group. ^†^ *p* < 0.05 compared with LGD-TVA group

### Alkaline sphingomyelinase enzymatic protein expression (Fig. 7)

In comparison with the C group there were no significant differences in alkaline smphingomyelinase enzymatic protein expression between the TA and LGD-TGA groups. However, a trend towards an increase in alk-SMase enzymatic protein expression in the TA group and a trend towards a decrease in alk-SMase enzymatic protein expression in LGD-TVA were found. On the other hand, the HGD-TVA group was characterized by significantly decreased alk-SMase enzymatic protein expression compared with the C group (*p* < 0.05), suggesting decreased alk-SMase activity in the HGD-TVA group. Further analysis revealed that expression of alk-SMase enzymatic protein was significantly decreased in both LGD-TVA and HGD-TVA in comparison with the TA group (*p* < 0.05 and *p* < 0.05, respectively), which suggests reduced alk-SMase activity in both the LGD-TVA and HGD-TVA groups. No difference in the expression of alk-SMase enzymatic protein between LGD-TVA and HGD-TVA groups was observed.

**Fig. 7:**
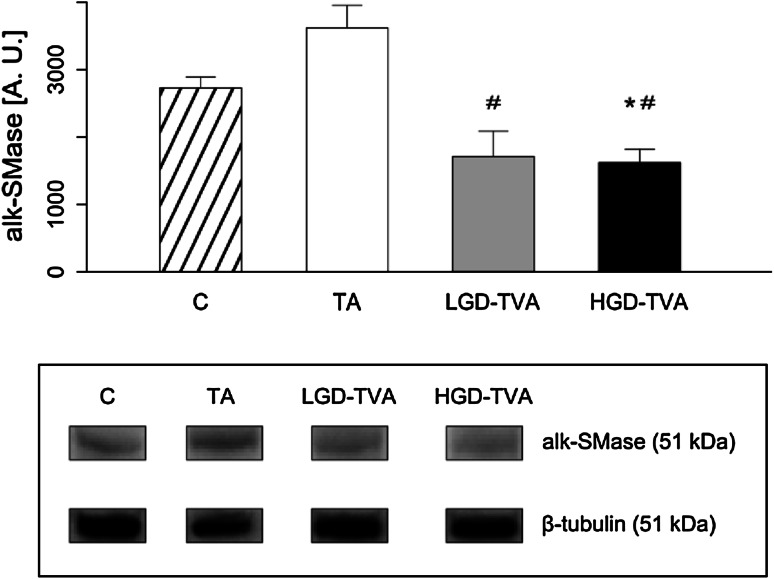
Comparison of alkaline sphingomyelinase (alk-SMase) enzymatic protein expression within all examined groups. *C* control group, *TA* tubular adenomas group, *LGD-TVA* tubulovillous adenomas with low-grade dysplasia group, *HGD-TVA* tubulovillous adenomas with high-grade dysplasia group. The results are mean ± SD; *p < 0.05 compared with C group;^ #^ *p* < 0.05 compared with TA group; ^†^ *p* < 0.05 compared with LGD-TVA group

### Sphingosine kinase 1 enzymatic protein expression (Fig. 8)

In comparison with the C group, sphingosine kinase 1 enzymatic protein expression was significantly decreased in both the TA and LGD-TVA groups (*p* < 0.05 and *p* < 0.05, respectively), which suggests reduction of SPHK1 activity in both the TA and LGD-TVA groups. On the contrary, the expression of described enzymatic protein was significantly increased in the HGD-TVA group (*p* < 0.05) suggesting upregulation of SPHK1 activity in the HGD-TVA group. The subgroup analysis showed that expression of SPHK1 enzymatic protein expression was significantly elevated in both LGD-TVA and HGD-TVA compared with the TA group (*p* < 0.05 and *p* < 0.05, respectively) suggesting increased activity of SPHK1 in both LGD-TVA and HGD-TVA. Finally, an increased SPHK1 enzymatic protein expression in the HGD-TVA group compared with the LGD-TVA group was noticed (*p* < 0.05), which suggests intensified SPHK1 activity in the HGD-TVA group.

**Fig. 8:**
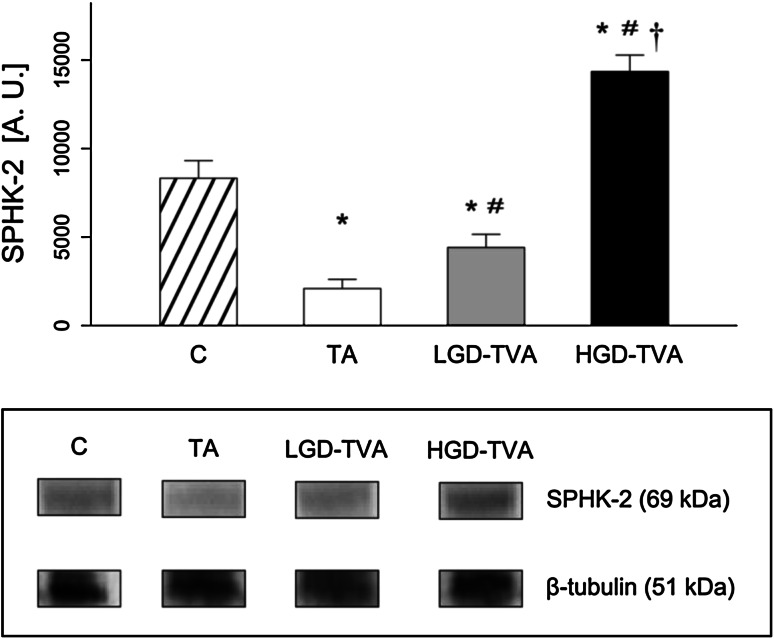
Comparison of sphingosine kinase 1 (SPHK1) enzymatic protein expression within all examined groups. *C* control group, *TA* tubular adenomas group, *LGD-TVA* tubulovillous adenomas with low-grade dysplasia group, *HGD-TVA* tubulovillous adenomas with high-grade dysplasia group. The results are mean ± SD. Results were back-transformed. *p < 0.05 compared with C group;^ #^ *p* < 0.05 compared with TA group; ^†^ *p* < 0.05 compared with LGD-TVA group

## Discussion

Although the incidence of colorectal cancer is continuously growing, the pathogenetic mechanism responsible for CRC’s onset and progression needs further clarification. It was postulated that one of the factors conditioning CRC development is abnormality in sphingolipid metabolism. Because colorectal adenomatous polyps are believed to be precancerous lesions with malignant potential, we have decided to evaluate disturbances in sphingolipids metabolism in cases of human CRAs. We have demonstrated, to our knowledge for the first time, changes in sphingolipid content followed by enzymatic activities which depend on histological architecture and grade of dysplasia of polypoid lesions.

The first important question is whether the histological state of polypoid lesions affects sphingolipid metabolism. Tubular adenomas are characterized by a relatively low risk of malignant transformation estimated at about 5 % [9]. In our studies, among 10 patients in the TA group we showed significant reduction of SFA content with a simultaneous decrease of SPT activity. These data clearly indicate that in cases of colon tubular adenomas, de novo ceramide synthesis pathway is inhibited. On the other hand, in the TA group ceramide level was significantly increased in comparison with healthy subjects. Developing our considerations further, SM concentration in the TA group was decreased in association with increased alkaline sphingomyelinase enzymatic protein expression. This could explain the increase of ceramide concentration in cases of tubular adenomas. In previously published studies, ceramide had been shown to stimulate apoptosis and inhibit cellular proliferation in CRC [26, 27]. Moreover, Selzner et al. demonstrated that ceramide level in human CRS samples was significantly decreased in comparison with healthy patients, confirming its protecting role in CRC progression [19]. Interestingly, measurement of ceramide derivatives concentration in the TA group in our study revealed that SPO content was higher while S1P level was significantly lower compared with the control group. Reduction in the concentration of S1P can be explained by a simultaneous decrease in sphingosine kinase enzymatic protein expression observed in the TA group. As mentioned above, S1P induces proliferation and inhibits apoptosis; therefore, it promotes neoplastic growth. Data published so far demonstrated also that S1P could stimulate cancer angiogenesis through the activation of platelet-derived growth factor (PDGF) and vascular endothelial growth factor (VEGF) [16]. In summary, it is noted that more malignant polypoid lesions are associated with lower ceramide and higher S1P contents.

On the other hand, villous component increases risk of malignant transformation by 20 % in cases of TVAs and even up to 40 % in cases of VAs [9]. In our study we have selected 10 subjects with low-grade dysplasia tubullovillous adenomas. In this group, SFA level, ceramide content, and SPT enzymatic protein expression were slightly elevated suggesting activation of ceramide de novo synthesis pathway in adenomas with villous component. Furthermore, we have noticed significant SM elevation, coexisting with decreased alk-SMase enzymatic protein expression. Data from the literature concerning this problem are limited, and so far only one work assessing the sphingolipid metabolism in cases of LGD-TVA was published. To investigate whether the changes in sphingolipid metabolism are present in the premalignant state of neoplastic transformation, Hertervig et al. [28] measured sphingomyelinases activities in patients with familial adenomatous polyposis and in sporadic colorectal TVAs [28]. In FAP adenoma tissue, activities of all SMase isoforms were markedly decreased. However, in sporadic TVAs (of which 70 % had low-grade dysplasia) only alk-SMase was significantly reduced (by 57 %) [28]. Other factors that may regulate adenomas progression are sphingosine kinase activity and S1P concentration. In our study we demonstrated that LGD-TAV group was characterized by decreased S1P level as a result of reduced SPHK1 enzymatic protein expression. In a previously published study Kohno et al. [22] demonstrated in the rodent model of FAP (APC^Min/+^ mouse) that knocking out of SPHK1 leads to reduction of S1P content followed by significant reduction of adenoma size [22]. On the contrary, in a group with the same histological architecture, but with high-grade dysplasia (HGD-TVA), S1P level was shown to be significantly elevated, suggesting that S1P concentration varies depending on severity of a grade of dysplasia rather than on presence of a villous component in adenoma histological architecture.

Colorectal adenomas with a villous component and high-grade dysplasia were considered to have the highest risk of malignancy [29]. Therefore, some researchers suggested that HGD-TVAs and HGD-Vas represent intramucosal carcinoma [30]. In our study we selected 11 patients with HGD-TVAs with characteristic cytological and architectural atypia, and we observed several changes in sphingolipid metabolism. First of all, in the HGD-TVA group SFA content was increased as well as SPT enzymatic protein expression, thus confirming previously noticed activation of a ceramide de novo synthesis pathway in adenomatous polyps with villous architecture. However, we noticed that total ceramide concentration in this group was significantly reduced in comparison with both healthy patients and groups with less advanced histological architecture or with a lower grade of dysplasia. Furthermore, alk-SMase enzymatic protein expression was also reduced simultaneously with an increase in SM content, suggesting inhibition of SM hydrolysis in cases of HGD-TVAs. Described alterations in sphingolipids metabolism in HGD-TVA group are in agreement with previously published changes in cases of CRC. Dudeja et al. [17] demonstrated in an animal model that SM level in colon cancer was increased [17]. Further studies by Dillehay et al. [18] proved that dietary SM (both natural from bovine milk and synthetic forms) supplementation ensured a relatively constant level of SM and ceramide in colonic mucosa and prevented formation of aberrant crypt foci by 70 %, inhibiting the development of CRC induced by chemical carcinogens [18]. Decreased ceramide level in colon cancer compared to healthy patients was also shown in human studies [19]. Presented changes in SM and ceramide contents are secondary to alterations in alk-SMase enzymatic protein expression. Moreover, it was demonstrated that alk-SMase activity was decreased by 75 % in human colorectal cancer [28]. In addition, as detected in stools of patients with CRC, alk-SMase was characterized by decreased activity compared to healthy patients [31], and abnormal isoforms of SMase expressed by colon cancer tissue are totally inactive [32]. The decrease of alk-SMase activity described in CRC and confirmed in our studies, in cases of precancerous lesions, HGD-TVAs reduced SM degradation and finally decreased ceramide formation [33]. Reduction of ceramide content in adenomatous polyps with high malignant potential may contribute to CRC development since ceramide can inhibit proliferative pathways together with activation of anti-proliferative and proapoptotic pathways in colorectal adenomas [34]. Furthermore, we found significant elevation in ceramide derivatives SFO and S1P in the HGD-TVA group (as a result of increased sphingosine kinase enzymatic protein expression). Similar changes were previously described in cases of colorectal cancer. Increased S1P content was observed in both human CRC and in animals treated with carcinogenic azoxymethane [20]. S1P acts through G-protein coupled receptors, localized on colonic epithelium plasma membrane, whereas Müller et al. [35] showed the intense upregulation of those receptors in human colon cancer cells [35]. Interestingly, by using specific anti-S1P antibodies, inhibition of growth, invasion, and angiogenesis in multiple tumor lineages, including colon cancer, can be obtained [21]. Therefore, as revealed in our study, increase in S1P concentration in the HGD-TVA group can be regarded as a potential factor responsible for unrestrained cell proliferation and neoplastic transformation of adenomatous polyps with advanced histology.

Although measurement of carcino-embryonic antigen plasma level is not reliable for diagnosis or as a screening for detection of CRC, its concentration correlates with disease stage and can be used to identify recurrences after surgical resection or to monitor occurrence of metastasis [36, 37]. Surprisingly, in our studies we observed elevated CEA level in the HGD-TVA group in comparison with healthy subjects. This finding suggests that adenomatous polyps with high malignant potential should be considered as early intramucosal carcinomas.

In conclusion, our study demonstrated, for the first time, significant changes in sphingolipid metabolism in different histological types of colorectal adenomatous polyps. On the basis of obtained data, we conclude that in cases of polypoid lesions with low malignancy potential, concentration of ceramide with proapoptotic and anti-proliferative properties increases, whereas anti-apoptotic S1P content decreases. These changes could potentially prevent transformation of tubular adenomas into polyps with higher malignancy potential. On the contrary, in the case of more advanced forms of adenomatous polyps, ceramide level is decreased while S1P concentration is increased, similarly as in the case of colorectal carcinoma. It is tempting to speculate that modulation of sphingolipid metabolism, leading to maintenance of constant ceramide level and decreasing of S1P concentration, could potentially inhibit the adenoma-carcinoma transformation pathway and reduce the incidence of CRC in the future.

## Abbreviations

alk-SMase: Alkaline sphingomyelinase
CEA: Carcino-embryonic antigen
CRA: Colorectal adenoma
CRC: Colorectal cancer
FAP: Familial adenomatous syndrome
GLC: Gas–liquid chromatography
HDL: High-density lipoprotein
HGD-TVA: Tubulovillous adenomas with high-grade dysplasia
HPLC: High performance liquid chromatography
LGD-TVA: Tubulovillous adenomas with low-grade dysplasia
PDGF: Platelet derived growth factor
S1P: Sphingosine-1-phosphate
SD: Standard deviation
SFA: Sphinganine
SFO: Sphingosine
SM: Sphingomyelin
SMase: Sphingomyelinase
SPHK: Sphingosine kinase
SPT: Serine palmitoyltransferase
TA: Tubular adenoma
TG: Triacylglycerol
TLC: Thin-layer chromatography
TVA: Tubulovillous adenoma
VA: Villous adenoma
VEGF: Vascular endothelial growth factor
WHO: World Health Organization
